# Influence of *IL-6*, *IL-8*, and *TGF-*β*1* gene polymorphisms on the risk of human papillomavirus-infection in women from Pernambuco, Brazil

**DOI:** 10.1590/0074-02760160051

**Published:** 2016-10-24

**Authors:** Sérgio Ferreira de Lima, Mayara Mansur Fernandes Tavares, Jamilly Lopes de Macedo, Renata Santos de Oliveira, Sandra de Andrade Heráclio, Maria de Mascena Diniz Maia, Paulo Roberto Eleutério de Souza, Ronald Moura, Sergio Crovella

**Affiliations:** 1 Universidade Federal de Pernambuco, Pós-Graduação em Biologia Aplicada à Saúde, Laboratório de Genoma, Recife, PE, Brasil; 2 Rede Nordeste de Biotecnologia, Recife, PE, Brasil; 3 Universidade Federal Rural de Pernambuco, Recife, PE, Brasil; 4 Instituto de Medicina Integral Professor Fernando Figueira, Recife, PE, Brasil; 5 Universidade Federal Rural de Pernambuco,Departamento de Biologia, Recife, PE, Brasil; 6 Universidade Federal de Pernambuco, Departamento de Genética, Recife, PE, Brasil

**Keywords:** polymorphisms, cytokine, host genome, HPV

## Abstract

Human papillomavirus (HPV) infections are strongly associated with the development of cervical intraepithelial neoplasias and invasive cervical cancer. Polymorphisms in cytokine-encoding genes and behavioural cofactors could play an important role in protecting an individual against viral infections and cancer. Here, we investigated whether *IL-6* -174 G>C, *IL-8* +396 G>T, and *TGF-β1* +869 G>C and +915 G>C polymorphisms were associated with susceptibility to HPV infection in women from north-east (Pernambuco) Brazil. We analysed 108 healthy uninfected women (HC) and 108 HPV-positive women with cervical lesions. Genetic polymorphisms were assessed using Sanger sequencing and polymerase chain reaction-restriction fragment length polymorphism. Comparison of the distribution of the genotypic and allelic frequencies of the IL-18 +396 T>G polymorphism between HPV infected woman an uninfected controls showed that the GG genotype and G allele were both more frequent in the HC group, and were associated with protection from HPV infection (p = 0.0015; OR = 0.29 CI95% = 0.13-0.61; p = 0.0005; OR = 0.45 CI95% 0.29-0.7, respectively). Individuals from the control group could have previously had HPV infection that was spontaneously eliminated; however, it was undetectable at the time of sample collection. Based on our findings, we hypothesize that the *IL-8* +396 G>T polymorphism could interfere with susceptibility to HPV infection, by modulating the ability of immune system to fight the virus.

Cervical cancer represents a significant public health problem causing a great impact worldwide; every year approximately 528,000 new cases and 266,000 deaths (7.5% of all female cancers) are expected (IARC 2012). In Brazil, cervical cancer is the second most common type of cancer among women. Approximately 16,340 new cases are expected for 2016, representing 15.85 cases per 100,000 individuals (INCA 2016). Infections with oncogenic types of the human papillomavirus (HPV) are responsible for most cases of cervical cancer and precancerous intraepithelial lesions (Muñoz et al. 2003).

Although the incidence of genital HPV infections is high, most of these infections are transient and do not lead to cervical intraepithelial neoplasia (CIN) or cancer, suggesting that other factors such as the immune system as well as the host genetic background could influence disease risk (Wu & Levine 1994).

HPV infection promotes immune cell migration to the dermis. In the squamous epidermis, macrophages, Langerhans cells (LC), T lymphocytes, dendritic cells (DC), natural killer cells (NK), and B-lymphocytes play important roles in the immune response to infection. HPV infection could induce the immune system to become more tolerant to the infection, thereby creating a microenvironment susceptible to further infection, facilitating CIN progression (Song et al. 2015).

These local immune responses seem to play an important role in the natural history of HPV infection of the uterine cervix. Cytokines are important regulators of HPV transcription due to their important role in the defence against HPV infection, through modulating viral replication (zur Hausen 2002). Polymorphisms in genes related to immunity have been reported to influence susceptibility to several diseases including viral infections. Therefore, these polymorphisms should also be considered when analysing the genetic susceptibility to HPV infection as well as cervical cancer (Wang & Hildesheim 2003).

The discovery of a TH17 variant has improved the understanding of inflammatory processes. TH17 cells are linked to chronic neutrophilic inflammation and can be induced to differentiate by transforming growth factor β1 (TGF-β1) and interleukin-6 (IL-6). Therefore, TGF-β1 and IL-6 can also induce the differentiation of TH9 and TH22 cells, respectively (both linked to tissue inflammation) (Akdis et al. 2011).

The IL-6 gene, located on the short arm of human chromosome 7 (7p21), presents several single nucleotide polymorphisms (SNPs), one of which is localised in the promoter region (-174G>C, rs1800795), and is associated with variations in *IL-6* expression and on serum protein levels (Fishman et al. 1998). This SNP correlates with poor prognosis in gastric cancer (de Vita et al. 2001) and prostate cancer (Nakashima et al. 2000). Moreover, IL-6 seems to be involved in cervical cancer progression and metastasis (Kinoshita et al. 1999, d, de Vita et al. 2001).

TGF-β1, a fundamental molecule involved in the homeostasis between cellular growth and apoptosis, is encoded by the *TGF-β1* gene located on the long arm of human chromosome 19 (19q13). The +869 T>C (rs1982073) SNP is located at codon 10 of exon 1 and results in a leucine-to-proline substitution, whereas +915 G>C (rs1800471) is located at codon 25 and results in an arginine-to-proline change. The C variant allele of codon 10, as well as the wild-type G allele of codon 25, is associated with increased production of TGF-β1 (Dunning et al. 2003). Furthermore, circulating levels of TGF-β1 have been associated with several diseases, including cancer (Elliott & Blobe 2005).

Interleukin-8 (IL-8), the first chemokine discovered, has pro-inflammatory activity, and is produced as part of immune or acute inflammatory reactions, as well as during chronic inflammation; this cytokine functions to attract and activate neutrophils in inflammatory regions (Campa et al. 2005). The *IL-8* gene is located on the long arm of chromosome 4 (4q12-q21) and several promoter, intron, and 3′UTR SNPs have been identified (Hull et al. 2001). The *IL-8* +396 G>T (rs2227307) polymorphism has been associated with respiratory diseases caused by viruses (Hull et al. 2001) and gastric cardiac adenocarcinoma (Savage et al. 2004).

Since few studies have been performed correlating SNPs in *IL-6*, *IL-8*, and *TGF-β1* genes with the risk of HPV infection, we evaluated whether *IL-6* (-174 G>C), *IL-8* (+396 G>T), and *TGF-β1* (+869 T>C and +915 G/C) functional genetic variants were related to HPV susceptibility, by studying HPV infected and uninfected women from Pernambuco, Brazil.

## SUBJECTS, MATERIALS AND METHODS

*Patients* - A hospital-based cross-sectional study was performed aimed at analysing *IL-6, IL-8*, and *TGFβ1* gene polymorphisms in HPV-infected women with cervical lesions and invasive cervical cancer (ICC). One hundred and eight women aged 17-68 years, with a mean age of 33.9 ± 10.1 years, presenting with either low-grade squamous intraepithelial lesion (LSIL) or high-grade squamous intraepithelial lesion (HSIL), were selected from the outpatient clinics of the Lower Genital Tract Pathology Clinic at the Women’s Healthcare Centre of the Prof Fernando Figueira Institute of Integrated Medicine, (Recife, Pernambuco, Northeast Brazil). Patients were selected by spontaneous demand from January 2009 to December 2010. Patients were included in the study if there was no discrepancy between cytological abnormality and histological diagnosis made at the first visit. All patients were initially assessed by colposcopy and subsequently cervical smears were collected. Histological diagnosis was made according to the Associação Brasileira de Genitoscopia (ABG 2002). Subjects were evaluated for clinical features of other sexually transmitted infections (STIs) based on history and examination. Patients who were previously submitted to radiotherapy or chemotherapy for ICC were excluded. Patients were also stratified according to age, parity, number of partners, smoking, and alcohol consumption.

One hundred and eight unrelated women from Pernambuco, enrolled at the Women’s Healthcare Centre, the same as that of HPV infected patients, aged between 14-66 years (mean age 37.2 ± 10 years), with no history of lesions or neoplastic disease as evaluated by the physician, and testing negative for HPV infection, were used as controls.

Informed written consent was obtained from the women, and the women were informed of the background of the study, risks and benefits, and voluntary nature of participation (CEP/CCS/UFPE Nº 355/08).

*Clinical samples* - Cervical smears were obtained using cytobrushes. Each cytobrush was packed in a TE buffer solution (10 mM Tris-HCl and 1 mM EDTA, pH 8.0) and maintained at -20ºC until analysis.

*DNA extraction* - Genomic DNA extraction was performed from 300 µL of cervical smear, from each study subject, using the Wizard Genomic DNA Purification kit (Promega, Madison, WI, USA) following the manufacturer’s instructions. The analyses were executed in Laboratory of Genetic, Biochemistry and DNA Sequencing (LGBS) of Rural Federal University of Pernambuco.

*HPV DNA detection* - Human papillomavirus DNA was detected from DNA extracted as previously described using two polymerase chain reaction (PCR) steps. The first was with MY09/11 external primers (MY09 5′-CGTCCMARRGGAWACTGATC-3′ and MY11 5′-GCMCAGGGWCATAAYAATGG-3′) and the second was with GP05+/06+ (GP5+ 5′-TTTGTTACTGTGGTAGATACTAC-3′ and GP6+ 5′-GAAAAATAAACTGTAAATCATATTC-3′) as internal ones. These two primers pairs are most widely used for the detection of genital HPVs. A negative control containing only digestion buffer was included for every five samples to prevent and detect carry-over between samples.

*IL-6, IL-8, and TGF-β1 SNPs genotyping* - *IL-6* and *TGF-β1* polymorphisms were amplified from the same DNA utilised for HPV detection, using specific primers (*IL-6* - 5′-TTGTCAAGACATGCCAAAGTG-3′ and 5′-TCAGACATCTCCAGTCCTATA-3′ and *TGF-β1 -* 5′-TTCCCTCGAGGCCCTCCTA-3′ and 5′-GCCGCAGCTTGGACAGGATC-3′) which flanked the polymorphisms. PCR was performed following standard protocols from the literature. Briefly, after amplification, the amplicons were submitted to a sequencing reaction using the DyEnamic ET Dye Terminator Cycle sequencing kit (GE Healthcare) according to manufacturer’s recommendations and were sequenced using a MegaBACE 1000 DNA Sequencer.

*IL-8* was amplified from the same DNA utilised for HPV detection using specific primers (5 ′-TAAAGGTTTGATCAATATAGA-3′ and 5′-CTTCCTTCTAATTCCAATATG-3′) according to literature, and the genotypes were identified through restriction fragment analysis polymerase chain reaction*-*restriction fragment length polymorphism (PCR-RFLP) using the *ScrFI* restriction enzyme. Thirty per cent of the samples were sequenced to confirm the results.

*Statistical analysis* - Univariate statistical analysis was performed using BioEstat 5.0 software. The study was cross-sectional, with independent samples consisting of nominal data (genotypes). The influence of polymorphism on the risk for development of (pre) neoplastic cervical disease was estimated by an odds ratio (OR) and a 95% confidence interval (CI). Allele frequencies were estimated by direct counting. Comparison between genotypic frequencies of patients and control groups was performed using a χ2 test; a Fisher’s exact test was used to compare the allele frequencies in contingency tables. The OR and their respective 95% CI values were determined. All p values ≤ 0.05 were considered statistically significant.

## RESULTS

Selected characteristics, including age, age of first sexual coitus, parity, smoking status, alcohol consumption, and oral contraceptive use of 108 HPV infected women with cervical lesions are summarised in Table I; 44 women (40.74%) were over 35 years of age; 73 (67.6%) were younger than 18 at the time of first sexual intercourse; 23 (21.3%) had a parity of five or more; 64 (59.3%) women reported regular use of alcohol; 41 (37.9%) women were smokers, and 55 (50.9%) women reported the use of oral contraceptives. There was no significant difference between women with HSIL and LSIL in terms of age of first sexual intercourse (p = 0.3787), parity (p = 0.5637), smoking (p = 0.8130), alcohol consumption (p = 0.9781), and oral contraception use (p = 0.9247). HSIL and LSIL were more frequent in subjects aged ≥ 35 years than in subjects < 35 years of age (p = 0.0124) (Table I).

**TABLE I:** Associations of clinical characteristics between human papillomavirus infected women with high-grade squamous intraepithelial lesions (HSIL) and those with low-grade squamous intraepithelial lesions (LSIL)

	LSIL	HSIL	OR	95%CI	P
Age					
< 35	18 (85.7%)	46 (52.9%)	1		
≥ 35	3 (15.3%)	41 (47.1%)	5.35	1.47 - 19.48	0.0124*
AFSI					
> 18 years old	9 (42.8%)	26 (29.9%)	1		
< 18 years old	12 (57.2%)	61 (70.1%)	1.76	0.66 - 4.68	0.3787
Parity					
< 5 births	18 (85.7%)	67 (77%)	1		
≥ 5 births	3 (15.3%)	20 (23%)	1.79	0.48 - 6.70	0.5637
Smoking					
No	14 (66.7%)	53 (60.9%)	1		
Yes	7 (33.3%)	34 (39.1%)	1.28	0.47 - 3.50	0.8130
Alcohol consumption					
No	8 (38.1%)	36 (41.4%)	1		
Yes	13 (61.9%)	51 (58.6%)	0.87	0.33 - 2.32	0.9781
OCP use					
No	11 (52.4%)	42 (48.3%)	1		
Yes	10 (47.6%)	45 (51.7%)	1.18	0.45 - 3.06	0.9247

AFSI: age of first sexual intercourse; OCP use: oral contraceptive use; OR: odds ratio; 95%CI: 95% confidence interval; P: p-value; *: statistically significant.

Table II shows the distribution of genotypes and allele frequencies of *IL-6* (-174 G>C), *IL-8* (+396 T>G), and *TGF*-*β1* (+869 G>C) and (+915 G>C) polymorphisms in HPV infected women and healthy uninfected controls. *IL6* and *TGF*-*β1* (+869 T>C) and (+915 G>C) polymorphism frequencies were in Hardy-Weinberg equilibrium in HPV infected women and uninfected controls, whereas the *IL-8* +396 T>G polymorphism showed frequencies not in agreement with the Hardy-Weinberg equilibrium in the two groups of subjects analysed.

**TABLE II:** Allele and genotype frequencies of *IL-6* (-174 G>C), *IL-8* (+396 G>T), and *TGF*-*β1* (+869 G>C) and (+915 G>C) polymorphisms among human papillomavirus (HPV) positive women and healthy uninfected controls from Pernambuco state (Northeast Brazil)

	HPV infected women	Healthy uninfected controls	OR (CI 95%)	P value
IL6				
Allele	n = 216 (Freq)	n = 216 (Freq)		
G	172 (0,8)	163 (0,75)	Ref	
C	44 (0,2)	53 (0,25)	0,79 (0,5 - 1,24)	0,3563
Genotype	n = 108 (Freq)	n = 108 (Freq)		
G/G	67 (0,62)	64 (0,59)	Ref	0,2027
G/C	38 (0,35)	35 (0,32)	1,04 (0,58 - 1,84)	0,9829
C/C	3 (0,03)	9 (0,08)	0,32 (0,08 - 1,23)	0,152
IL8				
Allele	n = 216 (Freq)	n = 216 (Freq)		
T	174 (0,81)	141 (0,65)	Ref	
G	42 (0,19)	75 (0,35)	0,45 (0,29 - 0,7)	0,0005*
Genotype	n = 108 (Freq)	n = 108 (Freq)		
T/T	77 (0,71)	66 (0,61)	Ref	0,0003*
T/G	20 (0,19)	9 (0,08)	1,9 (0,81 - 4,47)	0,1964
G/G	11 (0,1)	33 (0,31)	0,29 (0,13 - 0,61)	0,0015*
TGF-β1+869T/C				
Allele	n = 216 (Freq)	n = 216 (Freq)		
T	131 (0,61)	131 (0,61)	Ref	
C	85 (0,39)	85 (0,39)	1 (0,68 - 1,47)	0,9215
Genotype	n = 108 (Freq)	n = 108 (Freq)		
T/T	40 (0,37)	37 (0,34)	Ref	0,6905
T/C	51 (0,47)	57 (0,53)	0,83 (0,46 - 1,49)	0,628
C/C	17 (0,16)	14 (0,13)	1,12 (0,49 - 2,59)	0,9528
TGF-β1+915G/C				
Allele	n = 216 (Freq)	n = 216 (Freq)		
G	210 (0,97)	207 (0,96)	Ref	
C	6 (0,03)	9 (0,04)	0,66 (0,23 - 1,88)	0,5992
Genotype	n =n108 (Freq)	n = 108 (Freq)		
G/G	102 (0,94)	99 (0,92)	Ref	
G/C	6 (0,06)	9 (0,08)	0,65 (0,22 - 1,89)	0,5924
C/C	0 (0)	0 (0)		

CI95%: 95% confidence interval; OR: Odds ratio; Ref: reference Allele; *: statistically significant.

*IL-6* -174 G>C and *TGF*-*β1* +869 T>C and +915 G>C polymorphism alleles and genotype frequencies were not statistically different between HPV infected women and healthy uninfected controls. Comparison of the *IL-8* +396 T>G polymorphism allele and genotype frequencies between HPV infected woman and uninfected controls showed a statistically significant increase in the frequency of the GG genotype in uninfected subjects compared to HPV infected women, suggesting an association between this allele and protection from HPV infection (p = 0.0015; OR = 0.29 CI95% 0.13-0.61). In addition, the prevalence of the G allele was more frequent in HCs than in HPV infected women, thus, suggesting an association between this allele and protection from HPV infection (p =0.0005; OR = 0.45 CI95% 0.29-0.70).

In addition, with the aim of comparing the allele frequencies of the SNPs observed in our study with those of the population from Pernambuco, we inferred the allele frequencies (indicated as f in the equation below; p indicates proportion) for each polymorphism based on the equation presented by Suárez-Kurtz et al. (2014):


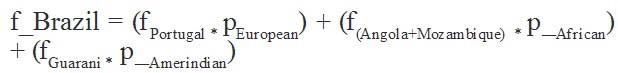


Essentially, this equation calculates a weighted average of the allele frequency observed in the parental populations of Brazil, considering the proportion of each ancestry found in the Brazilian population. Since we had no data regarding the populations described in the equation, we used the findings of some related populations with available data present in the 1000 Genomes Project database (http://browser.1000genomes.org) as proxies.

For each polymorphism, we obtained the allele frequencies from European Iberian (IBS), African Yoruba (YRI), and Peruvian South American (PEL) genome databases and combined the frequencies considering the admixture of the Pernambuco population, comprising genomes resulting from European (59.7%), African (23%), and Amerindian (17.3%) ancestries, as described in Coelho et al. (2015).

The predicted admixed frequencies for each polymorphism were as follows: 0.22 for the *IL-6* (-174 G>C) polymorphism; 0.41 for the *IL-8* (+396 T>G) polymorphism; 0.46 for *TGF*-*β1* (+869 T>C); 0.03 for the *TGF*-*β1* (+915 G>C) SNP. Finally, no significant differences in the distribution of *IL-6* (-174 G>C), *IL-8* (+396 T>G), *TGF*-*β1* (+869 T>C), and (+915 G>C) polymorphisms were observed between LSIL and HSIL groups (Table III) in HPV infected women.

**TABLE III:** Association of *IL-6* (-174 G>C), *IL-8 (*+396 T>G), and *TGF*-*β1* (+869 G>C) and (+915 G>C) polymorphisms among human papillomavirus (HPV) positive women with high-grade squamous intraepithelial lesions (HSIL) and those with low-grade squamous intraepithelial lesions (LSIL)

	LSIL	HSIL	OR	95%CI	P		LSIL	HSIL	OR	95%CI	P
IL-6						TGF-β1+869 T/C					
GG	13 (61.9%)	54 (62.1%)		1		TT	11 (52.4%)	29 (33.3%)		1	
GC	7 (33.3%)	31 (35.6%)	1.07	0.38 - 2.95	0.8923	TC	8 (38.1%)	43 (49.5%)	2.04	0.73 - 5.68	0.2343
CC	1 (4.8%)	2 (2.3%)	0.48		0.8827	CC	2 (9.5%)	15 (17.2%)	2.84	0.56 - 14.53	0.3420
G	33 (78.6%)	139 (79.9%)	1			T	30 (71.4%)	101 (58.1%)		1	
C	9 (21.4%)	35 (20.1%)	0.92	0.40 - 2.11	0.9811	C	12 (28.6%)	73 (41.9%)	1.81	0.87 - 3.76	0.1564
IL-8						TGF-β1+915 G/C					
GG	1 (4.8%)	10 (11.5%)		1		GG	20 (95.2%)	82 (94.2%)		1	
GT	8 (38.1%)	12 (13.8%)	0.15	0.02 - 1.41	0.1614	GC	1 (4.8%)	5 (5.8%)	1.22	0.13 - 11.03	0.7235
TT	12 (57.1%)	65 (74.7%)	0.54	0.06 - 4.63	0.9096	CC	0 (0%)	0 (0%)		nd	
G	10 (23.8%)	32 (18.4%)	1			G	41 (97.6%)	169 (97.1%)		1	
T	32 (76.2%)	142 (81.6%)	1.39	0.62 - 3.11	0.5625	C	1 (2.4%)	5 (2.9%)	1.21	0.14 - 10.67	0.7273

OR: odds ratio; 95%CI: 95% confidence interval; P: p-value.

## DISCUSSION

Since HPV infection is a multifactorial process, depending on environmental and host genomic factors, with particular focus on immunoregulatory genes, we analysed the possible impact of genetic variants, namely *IL-6* (-174 G>C), *IL-8* (+396 T>G), and *TGF*-*β1* (+869 T>C) and (+915 G>C) (cytokines encoding genes), on the susceptibility to HPV infection in women (HPV infected and uninfected) from Pernambuco (Brazil).

We did not find any significant association between the *IL-6* +174 G>C polymorphism and HPV infected women or uninfected controls, or between HSIL and LSIL subgroups; we also did not find any relationship between *TGF-β1* +869 T>C and +915 G>C polymorphisms and the risk of HPV infection or the development of LSIL or HSIL. *IL-6* and *TGF-β1* polymorphisms were not associated with the development of cervical cancer in a population from Zimbabwe, Africa (Stanczuk et al. 2002), and in a population from Shaaxi, China (Wang et al. 2011). In Brazil, Fernandes et al. (2008) did not find any correlation between *TGF-β1* +869 G>C and +915 G>C polymorphisms and the risk for HPV-related cervical lesions in women from São Paulo. Marangon et al. (2013) reported similar findings in HPV infected women from Paraná, Brazil.

Analysis of the *IL-8* +396 T>G polymorphism revealed an increased frequency of the TT genotype in HPV infected women, whereas the GG genotype and the G allele were both more frequent in healthy uninfected women, and were thus associated with protection from HPV infection.

It is worth noting that both HPV and HC groups were not in Hardy-Weinberg Equilibrium for the *IL-8* +396 T>G SNP due to an excess of homozygosity when compared to the expected genotype frequencies predicted for the general population of Recife. Furthermore, the minor allele frequency of this polymorphism in both HPV (0.19) and HCs (0.35) was reduced in comparison to that predicted by ancestry estimates (0.41). Therefore, more studies are needed to investigate the lower than expected heterozygosity in this population.

IL-8, a chemokine, acts by attracting granulocytes to sites of inflammation, resulting in neutrophil-mediated viral clearance (Knall et al. 1997). IL-8 expression is primarily regulated by an activator protein and/or nuclear factor-κB-mediated transcriptional activity, which is a critical regulator of the immediate early pathogen response that acts in response to bacterial and viral infections (Brat et al. 2005).

IL-8 has been hypothesized to play an important role in ovarian cancer (Kassim et al. 2004). Increased IL-8 serum levels were related to HPV persistence (Baker et al. 2011); IL-8 has been also reported to be involved in the epithelial-mesenchymal transition and the tumour microenvironment (Palena et al. 2012). Some viruses such as Herpes Simplex Virus (HSV), Epstein-Barr Virus (EBV), Human Immunodeficiency Virus (HIV) and Human T Lymphotropic Virus (HTLV-1) have been shown to induce IL-8 expression through the Nuclear Factor-κB (NF-κB) pathway (Mogensen & Paludan 2001).

Differential expression of IL-8 could be associated with the presence of functional polymorphisms such as the *IL-8* +396 G/T. The *IL-8* +396 GG genotype is known to yield higher levels of IL-8 and has been linked to the development of ovarian carcinoma (Wang et al. 2012). Therefore, we hypothesize that individual carriers of the *IL-8* +396 GG genotype, associated with high levels of IL-8, are better able to control HPV infection through the attraction of granulocytes to the sites of infection. Consequently, this would results in successful neutrophil-mediated viral clearance. Nevertheless, this is a hypothesis that needs to be verified by analysing IL8 serum levels, was not possible to analyse in this study. Moreover, the *IL-8* +396 T/G polymorphism allele and genotype frequencies assessed in HPV infected and uninfected women were not in Hardy-Weinberg equilibrium and were different from those inferred for the Pernambuco population. Therefore, our findings should be considered with caution and thus need to be replicated in at least one other north-east Brazilian population.

We are also aware that the main limitation of this study is the relatively small cohort enrolled; furthermore, HPV high-risk and low-risk genotyping data were not available to us; therefore, it was impossible to evaluate the impact of HPV genotypes on the susceptibility to cervical lesions as well as their relationship with cytokine-encoding gene polymorphisms.

In conclusion, taking into account the fact that individuals from the control group could have been previously infected by HPV that was spontaneously eliminated, and based on our findings, we hypothesize that the *IL-8* +396 G allele and GG genotypes could play a role in the risk of HPV infection in our study population. Specifically, carriers of these genetic variants, responsible for higher IL-8 production, should be able to react better to HPV infection. This work will prompt future genetic studies regarding the molecular pathogenesis of HPV infection and similar analyses should be performed to determine whether this polymorphism could serve as a prognostic risk factor for HPV infection.
